# Resting State EEG Related to Mathematical Improvement After Spatial Training in Children

**DOI:** 10.3389/fnhum.2021.698367

**Published:** 2021-07-08

**Authors:** Da-Wei Zhang, Anna Zaphf, Torkel Klingberg

**Affiliations:** Department of Neuroscience, Karolinska Institutet, Stockholm, Sweden

**Keywords:** children, mathematical training, spatial training, transfer effect, EEG

## Abstract

Spatial cognitive abilities, including mental rotation (MR) and visuo-spatial working memory (vsWM) are correlated with mathematical performance, and several studies have shown that training of these abilities can enhance mathematical performance. Here, we investigated the behavioral and neural correlates of MR and vsWM training combined with number line (NL) training. Fifty-seven children, aged 6–7, performed 25 days of NL training combined with either vsWM or MR and participated in an Electroencephalography (EEG)-session in school to measure resting state activity and steady-state visual evoked potentials during a vsWM task before and after training. Fifty children, aged 6–7, received usual teaching and acted as a control group. Compared to the control group, both training groups improved on a combined measure of mathematics. Cognitive improvement was specific to the training. Significant pre-post changes in resting state-EEG (rs-EEG), common to both training groups, were found for power as well as for coherence, with no significant differences in rs-EEG-changes between the vsWM and MR groups. Two of the common rs-EEG changes were correlated with mathematical improvement: (1) an increase in coherence between the central frontal lobe and the right parietal lobe in frequencies ranging from 16 to 25 Hz, and (2) an increase in coherence between the left frontal lobe and the right parietal lobe ranging from 23 to 25 Hz. These results indicate that changes in fronto-parietal coherence are related to an increase in mathematical performance, which thus might be a useful measure in further investigations of mathematical interventions in children.

## Introduction

Early mathematical abilities predict not only later mathematical achievement but also general school performance (Duncan et al., 2007; Geary, 2011) and later socioeconomic status (Ritchie and Bates, 2013). Research into the cognitive and neural basis for mathematical abilities might provide one way of developing effective early mathematical interventions (e.g., Butterworth et al., 2011; Clements and Sarama, 2011; Duncan and Magnuson, 2013).

One of the potential cognitive bases for math performance is spatial abilities, such as mental rotation (MR) (Wai et al., 2009) and visuo-spatial working memory (vsWM) (Peng et al., 2016). These correlations in performance have led to suggestions of enhancing mathematical ability through spatial training (Stieff and Uttal, 2015). Training on spatial tasks has indeed enhanced mathematical performance in some studies of rotation training (Hawes et al., 2017; Lowrie et al., 2017; Mix et al., 2020; but see also Hawes et al., 2015) as well as for vsWM (Bergman-Nutley and Klingberg, 2014; Berger et al., 2020; Mix et al., 2020; Judd and Klingberg, 2021; but see also Roberts et al., 2016).

Mix et al. (2020) recently compared children training on either vsWM or MR tasks and found that both groups improved compared to a control group engaged in language training. There was no significant difference between the two training groups in terms of the amount of improvement, nor on the type of mathematical tasks that were improved. A recent study in more than 17,000 typically developing children compared training with either MR or vsWM (Judd and Klingberg, 2021). At baseline, performance on the vsWM and MR were highly correlated, and both were correlated with baseline tests of mathematics. Training led to a math improvement of 0.56 SD estimated with online tests, with children training on the vsWM improving slightly more than children practicing rotation, although the difference was small (about 0.05 SD).

Although informative of the relative benefits of vsWM and MR training, the studies by Mix et al. (2020) and Judd and Klingberg (2021) did not evaluate neural correlates of training. Here, we evaluated similarities and differences in the pre-post changes in resting state-Electroencephalography (rs-EEG) in the two training groups. rs-EEG has been suggested as a useful biomarker in pediatric clinical research (Loo et al., 2016) and could fill a similar role in educational neuroscience. We chose rs-EEG based on prior findings of association between rs-EEG and cognitive performance. For example, frontal and parietal spectral power from rs-EEG correlates with executive functions (Zhang et al., 2017, 2018; Perone et al., 2018; Cai et al., 2021) and IQ (Thatcher et al., 2005, 2007). In addition to rs-EEG spectral power, fronto-parietal connectivity measured by coherence has been suggested to be a useful measure related to executive functions and IQ (Thatcher et al., 2005; Barry et al., 2011; Fleck et al., 2016; Teramoto et al., 2016; Han and Chan, 2017). In particular, fronto-parietal connectivity (e.g., correlation of fMRI time-series) has been identified as an important aspect of changes during vsWM and mathematical improvement (Darki and Klingberg, 2015; Constantinidis and Klingberg, 2016; Nemmi et al., 2018). However, as it is difficult to hypothesize the frequency features of the training-induced changes, we used a data-driven approach to analyze spectral and coherence measures (Maris and Oostenveld, 2007).

In addition, the training effect was evaluated with a steady-state evoked potentials (SSVEP) task. SSVEP has a high signal to noise ratio (Vialatte et al., 2010; Norcia et al., 2015), which might make it useful in studies of younger children and has been recently used to evaluate numerical processing (Park, 2018). The technique capitalizes on a steady frequency of stimulation to elicit a cortical signal at the same frequency (Vialatte et al., 2010; Norcia et al., 2015). Here, we adapted SSVEP to a vsWM paradigm to assess the training effect.

Secondly, this study evaluated the transfer of vsWM or MR to non-trained vsWM or rotation tasks. Since vsWM and MR are positively correlated (Mix et al., 2018; Malanchini et al., 2020), and both types of training have had beneficial effects on mathematical performance, one might assume that there is some commonality in the neural and cognitive effects of these two types of training. Spatial training generally has medium transfer effects on the spatial ability differing from the trained one (Uttal et al., 2013). However, there is very limited evidence on the mutual transferability between vsWM and MR. Two studies in adults explored the question regarding the effect of vsWM training on MR, and both failed to find significant effects (Chooi and Thompson, 2012; Stephenson and Halpern, 2013). However, given the general transferability of spatial training reported in children (Uttal et al., 2013), we hypothesize that both MR training and vsWM training would show the mutual transfer. Here, we firstly evaluated transfer for MR training to vsWM performance and vice versa.

Lastly, we evaluated the effect sizes of these two types of spatial training combined with mathematical training on mathematical performance. A recent study suggests that different forms of spatial training combined with mathematical training have comparable effects on improving mathematics (Judd and Klingberg, 2021); however, the effect size was not estimated. Hence, we compared two combined training groups to a control group who received teaching as usual. The mathematical training was based on the usage of a number line (NL) since such training has previously been found to be effective (Fischer et al., 2011; Nemmi et al., 2016; Outhwaite et al., 2019). We thus expected both groups to improve in mathematics, and the aim was not to directly compare the improvements in mathematics between the two training groups since prior data (Mix et al., 2020; Judd and Klingberg, 2021) suggested that such differences are small, and the current study was underpowered to detect this.

## Materials and Methods

### Participant

Children were recruited from pre-school and first-grade classes at two schools in Stockholm. Training of vsWM and MR was randomized within classes. Two training groups consisted of 63 children. Six children dropped out because of sickness. A control group was recruited from a separate school, consisting of 48 children (two children dropped out because of sickness). The demographic information for each group is shown in Table 1. The school principals, teachers, and caregivers were informed about the study through email and consent to participate in this study. Written consents were obtained from the children and caregivers.

**TABLE 1:** Demographic information for each group.

**Group**	***n***	**Grade (preschool)**	**Gender (female)**	**Age (month)**	**Math hours**
Control	48	25	25	86 (7.4)	14.8 (4.6)
vsWM + NL	29	17	11	87 (7.0)	15.4 (4.6)
MR + NL	28	17	19	86 (6.8)	15.6 (4.6)

*Standard deviations are shown in brackets. Math hours refer to the amount of mathematical training in school between the pre and post-training.*

*vsWM = visual-spatial working memory; NL = number line; MR = mental rotation.*

### Procedure

This work was approved by the Swedish ethical committee in Uppsala (2018/2155-31 and 2019/06514). Each child completed cognitive and mathematical tests 1–2 weeks before the training. The tests were administered in a quiet room within the schools. When a child entered the room, a tester repeated information from the written consent. Once oral consent was obtained, each child was guided to complete three mathematical tests, a vsWM test, and an MR test. This took about 15–20 min. The children in the training groups attended an additional rs-EEG recording and an SSVEP task of vsWM after a short break. The same tests were administered after training.

The training was administered through an application on iPad. The children in training groups were asked to aim to do 25-session daily training at school. Each session took about 30 min. The training was delivered as a part of the daily curriculum, and the teachers were responsible for monitoring the training process. A research assistant attended the first few sessions to support the teachers. The control group attended school as usual but without the training.

Due to the impact of COVID-19, changes were made during the training. While the schools remained open during the crisis, some children preferred staying at home and were willing to continue their training plans. Hence, the children were provided with hardware and software to continue. Their parents were instructed to use the application.

### Training Program

**FIGURE 1:**
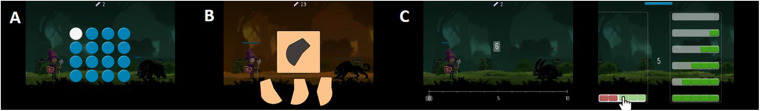
The screenshots for the training games: visual-spatial working training **(A)**, mental rotation training **(B)**, and mathematical training **(C)**.

The training was delivered via a free app -- Vektor -- from the non-profit foundation Cognition Matters^1^. Vektor aims to improve mathematical performance in children through enhancing number sense and cognitive abilities underpinning mathematical performance. Screenshots for different types of training in Vektor are shown in Figure 1. The vsWM + Math training group consisted of working memory training (50%) and mathematical training (50%). The MR + Math training group consisted of MR training (50%) and mathematical training (50%).

### Math Training

The mathematical training included both tasks using the numberline and a task called numberpals. During NL training, children needed to solve problems by dragging along an NL. At the beginning level, an NL was presented along with an Arabic number, and children were guided to use index fingers to drag the NL from zero to the correct position corresponding to the displayed Arabic number. After a few correct trials, the Arabic number was replaced by a mathematical problem of addition or subtraction. To solve addition problems, for example, 2 + 1, children were guided to drag along the NL from zero to 2 and then from 2 to 3. For subtraction problems, e.g., 2–1, children were guided to drag along the NL to 2 and then from 2 to 1. There was no time constraint for each trial.

Numberpals training was based on the ten-pal task (Butterworth et al., 2011). A bar with a length 0–10 was presented on the left side; meanwhile, ten different bars ranging from 0 to 10 were presented on the right side. The task was to drag the correct bar from the right side to the left side to get the sum of 10.

### Visuo-Spatial Working Memory Training

Visuo-spatial working memory training was conducted as previously described (Nemmi et al., 2016). Several similar vsWM tasks were presented, where dots or objects with different locations were presented on the screen in sequence. Children were expected to remember the sequence and to reproduce it by touching the screen. The difficulty level was adaptive by changing the number of cues.

### Mental Rotation Training

Mental rotation training consisted of problems in which children were expected to choose one out of three optional shapes to fit in a cut-out image. Children were guided to select the correct shape and to drag it into the cut-out part of the image. The difficulty was adaptive by increasing the degree to which the items were rotated and the complexity of the items.

### Behavioral Test

#### Mathematical Test

Three mathematical tests were administered: addition, subtraction, and verbal arithmetic. In the addition and subtraction tests, children were guided to answer arithmetic problems presented on paper by pointing at the number on cards ranging from 0 to 9. Answers larger than nine were responded by pointing twice. The test ended after 3 min or three incorrect answers in a row. Practice trials were given before formal testing.

A subtest from the WISC-V was used for testing verbal arithmetic. A tester read out arithmetic problems, and the children responded verbally. Each answer was required to be given within 30 s. The test ended after three incorrect answers in a row.

#### Visuo-Spatial Working Memory Test

The spatial span board from WISC-V was used for assessing the capacity of vsWM. Children were instructed to repeat the sequence of the positions which a tester just pointed at on a span board. There were 2 practice trials. The formal test started with 2 positions and increased with 1 more position each time. Children had 2 attempts at each level, and the test would stop until 2 failures at the same level.

#### Mental Rotation Test

A child-friendly MR test was used, consisting of 16 animal pictures presented on paper (Neuburger et al., 2011). At each trial, one exemplar picture is presented along with four rotated pictures. Only 2 of the four rotated pictures are identical to the exemplar picture, and the other two pictures are mirrored. The rotation angles consist of 45°, 90°, 135°, 225°, 270°, and 315°. Children are instructed to identify the two identical pictures and to answer as many trials as possible in 2 min.

### EEG Recording

**FIGURE 2:**
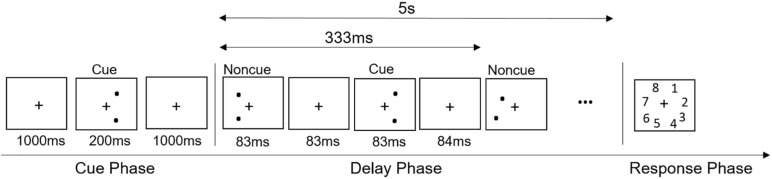
The task outline of the steady-state visual evoked potentials for working memory (SSVEP-vsWM).

EEG signals were acquired via a wireless EEG system with dry sensors (ENOBIO-32, Neuroelectrics). Eight positions of the 10–20 system were employed to record brain activity over frontal and parietal regions, including F3, Fz, F4, P3, Pz, P4, P7, and O1. Two extra channels were used to record the vertical electrooculogram of each eye. All electrodes were online referenced to the Common Mode Sense and the Driven Right Leg attached to the right earlobe. EEG was amplified with a bandpass of 0–125 Hz and was digitized online at a sampling rate of 500 Hz.

rs-EEG data were recorded during an eyes-closed resting state for 3 min. The SSVEP-WM task outline is shown in Figure 2. It consisted of 15 practical trials and 20 experimental trials. Each trial began with a cue phase in which the two dots to be remembered always presented at the right visual field (at least 4.5° to right of the central fixation) for 200 ms. Then, a delay phase was presented, during which the cue presented in the right visual field at 3 Hz for 5 s, and non-cues were presented in the left visual field at 3 Hz. At the response phase, the children were instructed to point out the position of the cue. The task lasted for about 3.5 min. Pilot tests were firstly conducted to assure that children were able to remember the item in the cue phase.

### Behavioral Measures

Because some children solved all mathematical problems within the time limit at the post-training testing – a “ceiling effect,” we calculated an efficiency measure by dividing the number of correct answers by the time spent. For synthesizing the efficiency measure from each math task, a composite score was calculated by averaging the *Z* score of each math task. The *Z* scores for both pre-and post-training were based on means and standard deviations at pre-training.

A “ceiling effect” also existed for the post-training MR testing. Hence, an efficiency measure was calculated, indicating the number of correct answers per minute. In contrast, as no one reached the “ceiling” of the vsWM testing, the maximum WM level was used directly.

### EEG Measure

EEG data were pre-processed in EEGLAB (Delorme and Makeig, 2004). All channels were off-line re-sampled at 250 Hz and bandpass filtered from 1 to 70 Hz with a 50 Hz notch filter. To exclude artifacts, we firstly applied the Artifact Subspace Reconstruction (Kothe and Makeig, 2013) and conducted a visual inspection to double-check. Eyes artifacts were rejected by Gratton’s correction (Gratton et al., 1983). Epochs containing activity beyond ±150 μv were excluded.

For rs-EEG, continuous EEG data were segmented into 2-s epochs. Hence, the frequency resolution in this study was 0.5 Hz. We calculated spectrum power and coherence between 1 and 25 Hz with a step of 0.5 Hz over the fronto-parietal channels. The process was done with functions in Fieldtrip (Oostenveld et al., 2011). A Fourier transform with a Hanning window was applied for quantifying spectral power at each channel. Coherence at a given frequency was calculated by the following equation:

$$C\,o\,h_{x\,y}\,\left(f\right)\,\frac{\left(G_{x\,y}\,{\left(f\right)}^{2}\right)}{\left(G_{x\,x}\,\left(f\right)\,G_{y\,y}\,\left(f\right)\right)}$$

where *G*_*xy*_(*f*) is the cross-spectral power between two electrodes and *G*_*xx*_(*f*) and*G_yy_*(*f*) are the two corresponding auto-spectral power. Coherence was calculated for the following 11 pairs: F3-P3, F3-Pz, F3-P4, Fz-P3, Fz-Pz, Fz-P4, F4-P3, F4-Pz, F4-P4, F3-F4, and P3-P4. Coherence values were transformed using Fisher’s *z*-transform.

For SSVEP-vsWM, the items to be remembered were presented in the right visual field. The analysis of SSVEP-WM focused on the contralateral occipito-parietal region. Specifically, this study quantified the response of electrodes over left posterior regions, including P3, P7, and O1, given that these regions show electrophysiological response during the vsWM delay phase (e.g., Vogel et al., 2005). In terms of frequency, as the response of SSVEP occurs at multiple harmonics of a stimulus frequency (Norcia et al., 2015), this study targeted at the cue-presenting frequency – 3 Hz – and its first two harmonics – 6 and 9 Hz. Signal-to-noise ratio (SNR) was calculated as the outcome and for each targeted frequency. In line with previous studies (Miskovic and Keil, 2015), SNR was quantified as the ratio of the target frequency power compared to ± 1 neighboring bands. A left posterior SSVEP (LP-SSVEP) was calculated by averaging the SNR across the targeted frequencies and then across the three left posterior electrodes.

### Statistical Analysis

The effects of the training protocols on the behavioral measures were examined by the residualized model (Castro-Schilo and Grimm, 2018) – the post-training score was predicted by the pre-training score, Group, age, and gender.

(1) “Post vsWM ∼ Pre vsWM + Group + Age + Gender.”
(2) “Post MR ∼ Pre MR + Group + Age + Gender.”

The days between the pre-training testing and the post-training testing varied between children, which resulted in that children received a different amount of formal math education between the gap. Given this may affect the post-training math score, math schooling hours were estimated by the product of the days and daily math learning duration. Thus, the residualized model for math was

(3) “Post math ∼ Pre math + Group + Age + Gender + Math schooling hour.”

In the case of a significant Group effect, the planned contrasts were conducted to compare each training group to the control, and the effect size was estimated by Cohen’s *d*.

The residualized model relies on the assumption that the factor of interest is not significantly correlated with covariates (Castro-Schilo and Grimm, 2018). Hence, we firstly examined if there were effects of Group on the other variable in the models.

The pre-post differences in rs-EEG power and coherence in the two training groups were examined by two-sided dependent *t*-tests with cluster-based permutations (Maris and Oostenveld, 2007). In the current study, the sample refers to power or coherence on each frequency, and clusters were formed by spectral adjacency. The significance of the sample-level comparisons was set as 0.01. At the cluster level, the analysis used the maximal sum of *t*-values and 10,000 iterations to detect significant clusters (*p* < 0.05). We also examined if the pre-post differences of EEG interacted with the training protocols. The analysis began with calculating the pre-post differences in the EEG measures for the two training groups. Then, the differences were examined by the same cluster permutation approach but with two-sided independent *t*-tests to simulate a 2 by 2 comparison.

The next step of the analysis was to examine if the pre-post EEG differences were associated with behavioral changes. The analysis was conducted by running bivariate correlations between the pre-post EEG differences that survived from the permutation tests and the pre-post differences of the behavioral tasks. The bias corrected and accelerated bootstrap 95% confidence intervals are reported together with Pearson’s *r*, estimated from 1,000 iterations.

The pre-post differences in SSVEP-vsWM were examined with the paired-samples *t*-test on LP-SSVEP.

## Results

### Demographic Information and Pre-test Profile

The demographic information for each group is shown in Table 1. Groups did not significantly differ in age (*F* = 0.314, *p* = 0.731), gender (*x*^2^ = 5.118, *p* = 0.077), or the math schooling hours (*F* = 1.768, *p* = 0.176). Also, there were no significant differences between the groups on the pre-tests (Math: *F* = 1.872, *p* = 0.159; vsWM: *F* = 1.296, *p* = 0.278; MR: *F* = 0.168, *p* = 0.845).

### Group Effect on Transfer Tasks

#### Mathematics Measure

**FIGURE 3:**
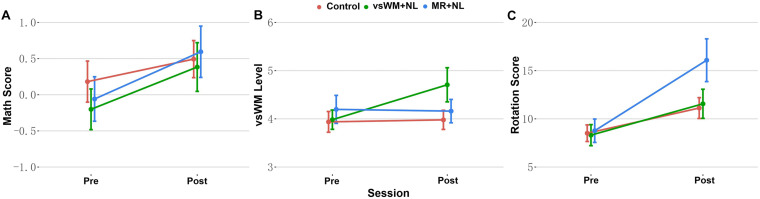
The pre-post performance of each group on mathematics **(A)**, visual-spatial working memory **(B)**, and mental rotation **(C)**.

There was a significant effect of Group on the post-math score (*F* = 5.972, *p* = 0.004, $\eta_{p}^{2}$ = 0.109), after controlling for age, gender, math schooling hours, and the pre-math score (Figure 3A). The planned contrasts indicated that both two training groups showed a higher post-math score compared to the control group (vsWM + NL vs. Control, *p* = 0.028, MR + NL vs. Control, *p* < 0.001). The results of the planned contrasts are summarized in Table 2.

**TABLE 2:** Results of the planned contrasts for each behavioral outcome.

	**Math**				**vsWM**				**MR**			
**Group**	**Mean_pre_**	**Mean_post_**	***p***	**Cohen’s *d***	**Mean_pre_**	**Mean_post_**	***p***	**Cohen’s *d***	**Mean_pre_**	**Mean_post_**	***p***	**Cohen’s d**
Control	0.18 (0.70)	0.26 (0.63)	–	–	3.9 (0.7)	4.0 (0.7)	–	–	8.5 (3.0)	11.1 (3.7)	–	–
vsWM + NL	−0.17 (0.55)	0.29 (0.58)	0.028	0.59	4.0 (0.5)	4.7 (0.9)	<0.001	1.02	8.3 (2.9)	11.6 (4.0)	0.444	0.22
MR + NL	−0.14 (0.60)	0.43 (0.61)	<0.001	0.74	4.2 (0.8)	4.2 (0.6)	0.606	−0.10	8.8 (3.1)	16.1 (5.7)	<0.001	1.55

*Standard deviations are shown in brackets. Math = the combined score (See Materials and Methods); vsWM = visual-spatial working memory; NL = number line; MR = mental rotation.*

#### Spatial Measure

Group had a significant effect (*F* = 9.123, *p* < 0.001, $\eta_{p}^{2}$ = 0.156) on the post-vsWM, after controlling for age, gender, and the pre-vsWM (Figure 3B). In the planned contrasts, compared to the control group, only the vsWM + NL group showed the improved post- vsWM (*p* < 0.001, *d* = 1.02), whereas the MR + NL group did not (*p* = 0.606, *d* = −0.10) (Table 2).

Group also showed a significant effect (*F* = 20.940, *p* < 0.001, $\eta_{p}^{2}$ = 0.297) on the post-test MR, after controlling for age, gender, and the pre-MR (Figure 3C). The planned contrasts showed that, compared to the control group, the MR + NL group showed the improved MR at the post test (*p* < 0.001, *d* = 1.55), and the vsWM + NL group did not (*p* = 0.444, *d* = 0.22).

### Pre–post EEG Change

**FIGURE 4:**
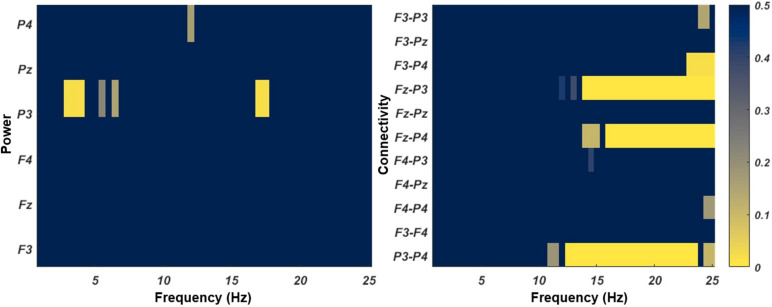
The rs-EEG clusters of spectral power and connectivity showed the Session effect. The color bar refers to the cluster-level *p*-values.

EEG analysis was conducted only in the two training groups. The results of cluster-based permutations for the effects of Session (pre-training vs. post-training) on power and coherence are shown in Figure 4. For rs-EEG spectral power, the two clusters at P3 that showed enhanced power at the post-test survived from the permutation test of the pre-post difference, one ranging from 3.0 to 4.0 Hz (*p* = 0.017) and the other one ranging from 17.0 to 17.5 Hz (*p* = 0.023). These power differences did not interact with the training protocols (vsWM + NL vs. MR + NL), i.e., these test–retest changes were similar in both training groups.

For rs-EEG coherence, four clusters showed increased coherence at the post: P3-P4 coherence ranging from 12.5 to 23.5 Hz (*p* = 0.001), Fz-P3 coherence ranging from 14.0 to 25.0 Hz (*p* = 0.002), Fz-P4 coherence ranging from 16.0 to 25.00 Hz (*p* = 0.006), and F3-P4 coherence ranging from 23.0 Hz to 25.0 Hz (*p* = 0.022). These coherence differences did not interact with the training protocols (vsWM + NL vs. MR + NL).

For SSVEP-vsWM, we only included the children who completed the tasks with an accuracy above 85%. Forty-seven children met the criterion. There was no Session effect on LP-SSVEP (*t* = 0.610, *p* = 0.545), i.e., no significant changes from pre- to post-training.

### EEG Change Correlated to Behavioral Change

**FIGURE 5:**
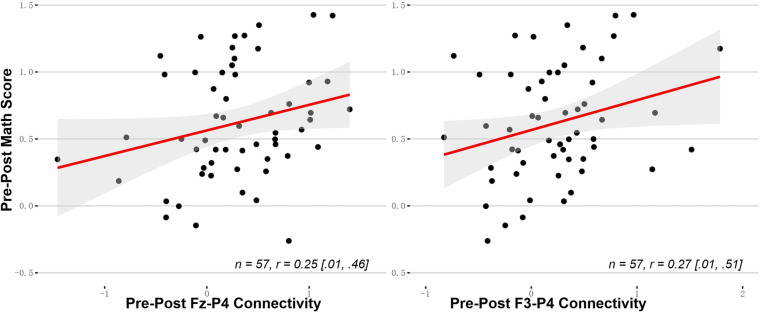
The fronto-parietal rs-EEG connectivity changes correlated with the mathematical improvement. The Bootstrap 95% confidence intervals are reported in the square brackets.

Given that there was no interaction between the EEG changes and the training protocols, we combined the two training groups to examine the relationship between the six EEG changes and the mathematical enhancement. Pearson’s *r* and the Bootstrap 95% confidence interval are reported. The mathematics enhancement was significantly correlated with the Fz-P4 change (*r* = 0.25, 95% Bca CI [0.01, 0.46]) and the F3-P4 change (*r* = 0.27, 95% Bca CI [0.01, 0.51]) (Figure 5).

As the two types of spatial enhancement were shown in different training groups, we examined the correlation of the EEG differences with the vsWM enhancement only in the vsWM + NL group and the MR enhancement only in the MR + NL group. There were no EEG changes correlated with the vsWM enhancement and the MR enhancement.

## Discussion

Here, we analyzed improvements in mathematical and spatial abilities in two training groups, compared to a control group. We found that both groups improved on the pre-post tests on a combined measure of mathematical performance, compared to our control group, which is in line with prior studies of this method (Nemmi et al., 2016; Judd and Klingberg, 2021). In contrast to our expectations, we did not find any significant improvement in an MR task after vsWM training or improvement on a vsWM task after MR training. We found two pre-post increases in coherence between the frontal lobe and right parietal cortex (Fz-P4 and F3-P4), which were correlated to the amount of improvement on the mathematical tests.

Both training groups showed improvements on the trained tasks: compared to the control group, vs-WM training and MR training improved on an untrained vs-WM task and an untrained MR task, respectively, which is in line with previous findings (Klingberg, 2010; Uttal et al., 2013; Yang et al., 2020). Our participants showed about one standard deviation improvement for each spatial training, compared to the test–retest improvements in the control group. The magnitude is close to the effect size of spatial training studies during early childhood (Hedges’s *g* = 0.96, Yang et al., 2020) but is higher than that of spatial training studies across ages (Hedges’s *g* = 0.47, Uttal et al., 2013), possibly in line with a suggestion that younger children may have a higher sensitivity to spatial training (Yang et al., 2020), although age was not found to moderate the effect of training in the meta-analysis by Uttal et al. (2013).

In contrast, the two spatial trainings did not show any transfer between the two cognitive tasks. The first explanation for this might be insufficient statistical power. A *post hoc* power calculation suggests that we had 80% power to detect an effect size of 0.69. However, a review estimated an effect size of 0.55 for transfer across different aspects of spatial tasks (e.g., from static spatial to dynamic spatial), and the effect of further transfer might be even smaller (Uttal et al., 2013). A second explanation is the outcome measures. Only a single MR task and a single vsWM task was used, and we cannot exclude that we might have found common cognitive effects using a broader test of spatial abilities that lower the task-specific aspects of the testing. Another possibility is that vsWM and MR tap different aspects of spatial ability (Hyun and Luck, 2007; Uttal et al., 2013; Mix et al., 2018). Although it has been argued that a common spatial ability explains most of the inter-individual variance (Mix et al., 2018; Malanchini et al., 2020), it could be different in young children (e.g., preschoolers, Mix et al., 2018).

Using an explorative analysis approach, we found three significant coherence changes after training, of which the middle frontal to right parietal coherence and the left frontal to right parietal beta coherence were correlated with mathematical improvement. Fronto-parietal EEG coherence has previously been associated with mathematical performance in cross-sectional studies. With rsEEG recording in children, the right fronto-parietal theta coherence is correlated with a mathematical composite score calculated from a range of counting and arithmetic problems (Anzalone et al., 2020); however, the study did not analyze the inter-hemispheric coherence. The correlation between fronto-parietal connectivity and mathematics was also reported for EEG recorded in other mathematical tasks. For example, the fronto-parietal coherence (González-Garrido et al., 2018) and frontal/parietal based coherence networks (Torres-Ramos et al., 2020) in a numerical comparison task are correlated with different levels of mathematical achievement in children, and the fronto-parietal power correlation in a logical-mathematical task is correlated with the behavioral outcome of the task in adults (Molina del Río et al., 2019).

An increase in fronto-parietal connectivity has also been proposed as one of the most important changes caused by working memory training measured during rest with imaging techniques (Jolles et al., 2013; Takeuchi et al., 2013; Constantinidis and Klingberg, 2016; Thompson et al., 2016). Here, we observed the fronto-parietal coherence increase with rs-EEG ranging from 12.5 to 25 Hz, after spatial training combined with NL training. The frequency range falls into the traditional beta band. This result is in line with the finding that beta coherence-based networks are enhanced after cognitive training (Klados et al., 2016). Given that beta activity is associated with top–down control (Engel and Fries, 2010), our result might indicate that the top–down control through fronto-parietal networks is enhanced after the combined training, which is consistent with imaging studies that fronto-parietal networks underlying top–down control tasks are enhanced after working memory training (Constantinidis and Klingberg, 2016). To our knowledge, this is the first study highlighting the possible brain changes in response to spatial combined with NL training. However, since we did not find any significant differences in rs-EEG between the training groups, and both groups performed mathematical training, the fronto-parietal coherence could not be specifically linked to either the MR or vsWM training but might be related to the mathematical training. Future studies, including more participants and more conditions, will be necessary.

To increase the feasibility of conducting this study in the school environment, we only employed a mobile system to record rs-EEG. Mobile EEG has been attracting substantial interests in developmental research due to that its flexibility eases the use of EEG in children (Lau-Zhu et al., 2019). One of its characteristic features is the significantly reduced amount of set-up time. This study further combined the feature with a low-density resting-state recording, a paradigm that does not require lengthy recording. On one hand, our results show the feasibility of a short-duration mobile EEG recording for school environment. On the other hand, the low-density recording does have limitations. The low spatial prevents us from more precisely localizing the anatomical and physiological basis for the EEG change. This might also be a reason why we did not observe EEG-changes specific to each of the two types of training.

Taken together, the present results prove the feasibility of EEG recordings in school, indicating that a short measurement of resting-state EEG can measure the behaviorally relevant change. In particular, our results suggest that fronto-parietal coherence might be a relevant measure for future studies of the impact of mathematical interventions.

## Data Availability Statement

The raw data supporting the conclusions of this article will be made available by the authors, without undue reservation.

## Ethics Statement

This work was approved by the Swedish Ethical Committee in Uppsala (2018/2155-31 and 2019/06514). Written informed consent to participate in this study was provided by the participants’ legal guardian/next of kin.

## Author Contributions

D-WZ and TK were responsible for the study concept and design. D-WZ and AZ contributed to the data collection and data analysis. All authors contributed to the article and approved the submitted version.

## Conflict of Interest

The authors declare that the research was conducted in the absence of any commercial or financial relationships that could be construed as a potential conflict of interest.

## Footnotes

1 https://cognitionmatters.org/
